# MRI-guided laser ablation for pediatric intracranial pathology: single center experience

**DOI:** 10.1007/s00381-026-07133-y

**Published:** 2026-01-24

**Authors:** Margaret P. Seaton, Muhammad S. Ghauri, Kiefer J. Forseth, Julia C. Schmidt, Ronald Sahyouni, Vijay M. Ravindra, David D. Gonda

**Affiliations:** 1 Department of Neurological Surgery, University of California, San Diego, San Diego, CA USA; 2 Loma Linda University Medical Center, Loma Linda, CA USA; 3 Department of Pediatric Neurological Surgery, University of Utah, Salt Lake City, UT USA; 4 Department of Pediatric Neurological Surgery, Rady Children Hospital, San Diego, CA USA

**Keywords:** Laser interstitial thermal therapy, Drug-resistant epilepsy, Minimally invasive neurosurgery

## Abstract

**Purpose:**

This study evaluates the safety and efficacy of MR-guided laser interstitial thermal therapy (MRgLITT) in 88 consecutive pediatric patients involving lesional and non-lesional epilepsy, brain tumors, cavernous malformations, and other intracranial masses.

**Methods:**

A retrospective case series was performed on pediatric patients who underwent MRgLITT by the senior author between 2016 and 2024. Demographic, intraoperative, and outcome data were analyzed.

**Results:**

Eighty-eight patients (47 males, mean age 14.0 years) were included. Indications comprised epilepsy (68 patients, 80 procedures), brain tumors (25 patients, 26 procedures), and cavernous malformations (8 patients, 8 procedures). Complications occurred in 12 of 88 patients (13.6%), including three major complications (3.4%). Among epilepsy patients, 35 of 68 (51.5%) achieved an ILAE 1 outcome. Among tumor patients, 17 of 25 (68%) had no recurrence at latest follow-up. CCM patients had favorable clinical courses; among those with follow-up MRI, 6 of 7 demonstrated no residual lesion. Seizure outcomes did not differ significantly between lesional and non-lesional epilepsy cohorts: ILAE 1 outcomes occurred in 28 of 49 (57.1%) lesional versus 7 of 19 (36.8%) non-lesional patients. Reoperation was more frequent in non-lesional patients (6 of 19, 31.6%), though ultimate outcomes were comparable (*p* = 0.17). On multivariable analysis, higher social vulnerability index predicted poorer seizure outcomes, highlighting the impact of social determinants on surgical results.

**Conclusions:**

MRgLITT demonstrated favorable safety and indication-specific outcomes for pediatric epilepsy, tumors, and cavernous malformations in this single-surgeon series. These findings support its role as both a primary and adjuvant treatment in pediatric neurosurgery.

**Supplementary Information:**

The online version contains supplementary material available at 10.1007/s00381-026-07133-y.

## Introduction

Magnetic resonance-guided laser interstitial thermal therapy (MRgLITT) is a minimally invasive neurosurgical procedure with broad utility in the treatment of adult epilepsy [1], tumors [2–4], radiation necrosis [5], and cerebral cavernous malformation (CCMs) [6]. MRgLITT, which was introduced in pediatric patients with contraindications to resection or with otherwise inaccessible lesions [7], is now widely used when open resection carries undue morbidity. Initial studies have demonstrated favorable outcomes in pediatric patients, and the use of MRgLITT has expanded to become a primary treatment option for lesional epilepsy [8] and select cases of intractable pathologies [9].

Minimally invasive treatment offers advantages over resection, including shorter hospital stays and preserved effectiveness in appropriately selected patients [10]. The most common application is in the treatment of drug-resistant epilepsy (DRE), where MRgLITT provides an initial treatment option with high seizure freedom rates [11] and neuropsychiatric and functional outcome preservation in both adult [12, 13] and pediatric [14] patients. Importantly, initial laser ablation does not preclude future ablation or resection should lesions be refractory to MRgLITT, thus providing an effective minimally invasive option that increases long-term management flexibility for pediatric patients [15]. With advancements in intraoperative guidance and ablation technology, MRgLITT has expanded to become an established treatment option for lesional epilepsy in pediatrics [16]; however, large-scale evidence detailing the role of MRgLITT in the management of pediatric non-lesional epilepsy [17], tumors [9, 18, 19], tuberous sclerosis [20], and CCMs remains limited.

Given the recent introduction of MRgLITT to pediatric neurosurgery, few applications are validated in large patient cohorts. The objective of this study is to address this gap by reporting on the safety and efficacy of MRgLITT in the treatment of 88 patients encompassing a variety of indications including epilepsy, tumors, and CCMs.

## Methods

A consecutive case series of pediatric patients who underwent MRgLITT for any indication via the Visualase Thermal Therapy System (Medtronic, Minneapolis, MN, USA) by the senior author between 2016 and 2024 was performed. All patients who underwent MRgLITT during the period with at least one follow-up visit were included. Epilepsy patients were excluded if they had less than 1 year of follow-up. Procedures were done using the ROSA robot for stereotactic navigation relative to preoperative MRI and CTA using either Leksell or Sugita frames. All research was completed at Rady Children’s Hospital (RCH) in San Diego, CA, with IRB approval. Human ethics and consent to participate declarations are not applicable.

Collected demographic data included gender, age, race, ethnicity, indication, stereo-encephalography (sEEG), BMI percentile for age, and social vulnerability index (SVI) calculated from patient zip code per CDC database [21]. The epilepsy cohort was subsequently split into lesional and non-lesional epilepsy based on the presence of an epileptogenic lesion on preoperative MRI. Patients with MRgLITT performed for the management of seizures secondary to tumors or cavernous malformations were included in both the lesional epilepsy subgroup and the tumor/CCM cohorts to enable epilepsy outcome analyses based on seizure presentation and MRI lesion status rather than underlying pathology. To improve interpretability, outcomes are reported by indication (epilepsy, tumor, CCM), and analyses were performed within each indication. Intraoperative data included the number of trajectories and the region ablated, while outcome data included complications, repeat ablation, seizure frequency percent change, International League Against Epilepsy (ILAE) outcome, length of follow-up, and lesion recurrence. We report demographics and seizure outcomes at the patient level while reporting procedural characteristics at the procedure level. For patients undergoing repeat ablation, seizure outcome was assigned using the outcome of the final procedure.

### Statistical methods

Demographic, intraoperative, and outcome data were collected from the medical record at RCH. Postoperative outcome was determined by the senior author (D.D.G.) at the final follow-up. The chi-squared test or the Fisher’s exact test was used to compare baseline characteristics between the three MRgLITT indications for ordinal variables, while the analysis of variance (ANOVA) test or independent sample *t*-test was used for continuous variables. Multivariable logistic regression was used to assess outcomes based on baseline demographic or perioperative variables. Statistics and figures were generated using scientific computing packages including NumPy, statsmodels, Matplotlib, and Seaborn in Python 3.12.7 [22].

## Results

### Population

A total of 88 patients (47 males (53.4%); mean age at surgery, 14.0 ± 6.6 years) underwent 100 MRgLITT procedures at RCH between June 2016 and October 2024. Demographic and perioperative data are presented in Table 1. Indications included lesional and non-lesional epilepsy (68 patients; 80 procedures), tumors (25 patients; 26 procedures), and cavernous malformations (8 patients; 8 procedures). Twelve patients underwent repeat ablation for inadequate symptom reduction.

**Table 1:** Clinical characteristics of included patients

Characteristic	Indication			Test used	*P* value
	Epilepsy	Tumor	Cav Mal		
Total patients, ***N*** (%)	68 (67.3)	25 (24.8)	8 (7.9)		
Male, ***N*** (%)	36 (52.9)	14 (56.0)	6 (75.0)	*X*^2^	0.49
Age at surgery, mean (SD), y	14.5 (6.8)	10.9 (6.0)	11.7 (6.2)	ANOVA	0.05
Race, ***N*** (%)				*X*^2^	0.84
White	56 (82.4)	18 (72.0)	8 (100.0)		
Black	4 (5.9)	3 (12.0)	0 (0.0)		
Asian	2 (2.9)	0 (0.0)	0 (0.0)		
AI/AN	1 (1.5)	0 (0.0)	0 (0.0)		
MENA	1 (1.5)	1 (4.0)	0 (0.0)		
Other	5 (7.4)	3 (12.0)	0 (0.0)		
Ethnicity				*X*^2^	0.55
Hispanic	29 (42.6)	8 (32.0)	4 (50.0)		
Non-Hispanic	39 (57.4)	17 (68.0)	4 (50.0)		
SVI, mean (SD)	0.60 (0.26)	0.55 (0.26)	0.47 (0.30)	ANOVA	0.32
Total procedures, ***N*** (%)	80 (70.2)	26 (22.8)	8 (7.0)		
Trajectories				*X*^2^	< 0.01
1	19 (23.8)	18 (69.2)	7 (87.5)		
2	38 (47.5)	5 (19.2)	1 (12.5)		
3	15 (18.8)	3 (11.5)	0 (0.0)		
4	5 (6.3)	0 (0.0)	0 (0.0)		
5	3 (3.8)	0 (0.0)	0 (0.0)		
Complications, ***N*** (%)	10 (13.2)	3 (11.5)	0 (0.0)	*X*^2^	0.36
Wound dehiscence	2 (20.0)	0 (0.0)	0 (0.0)		
Ataxia	0 (0.0)	1 (33.3)			
Transient weakness	7 (70.0)	1 (33.3)	0 (0.0)		
Bleed	1 (10.0)	1 (33.3)	0 (0.0)		
Hospital length of stay, mean (SD), d	2.3 (4.7)	1.3 (0.9)	1.0 (0.0)	ANOVA	0.54
Follow-up, mean (SD), y	3.4 (2.3)	3.3 (2.2)	3.4 (2.3)	ANOVA	0.89

The mean age of surgery for the epilepsy cohort was 14.5 ± 6.8 years; 36 patients were male (52.9%), 56 patients self-reported white race (82.4%), 29 patients were Hispanic (42.6%), and the average SVI was 0.60 ± 0.26 (Tables 2 and 3). Of the 80 epilepsy MRgLITT procedures, 55 (68.8%) were in lesional epilepsy patients by MRI, and 25 procedures (31.3%) were for treatment of non-lesional epilepsy. Among lesional patients, 16 procedures (29.1%) were performed for treatment of mesial temporal sclerosis (MTS) as defined by the ILAE [23], and 17 procedures (30.9%) occurred following pre-ablation sEEG. Mean length of follow-up in the epilepsy group was 3.4 years (range 1.0–8.2).

**Table 2:** Clinical outcomes of included patients per procedure

Characteristic	Indication			Test used	*P* value
	Epilepsy	Tumor	Cav Mal		
Total procedures, ***N*** (%)	80 (70.2)	26 (22.8)	8 (7.0)		
Trajectories				*X*^2^	< 0.01
1	19 (23.8)	18 (69.2)	7 (87.5)		
2	38 (47.5)	6 (23.1)	1 (12.5)		
3	15 (18.8)	3 (11.5)	0 (0.0)		
4	5 (6.3)	0 (0.0)	0 (0.0)		
5	3 (3.8)	0 (0.0)	0 (0.0)		
Complications, ***N*** (%)	10 (13.2)	3 (11.5)	0 (0.0)	*X*^2^	0.36
Wound dehiscence	2 (18.2)	0 (0.0)	0 (0.0)		
Transient weakness	7 (63.6)	1 (33.3)	0 (0.0)		
Ataxia	0 (0.0)	1 (33.3)	0 (0.0)		
Bleed	1 (18.2)	1 (33.3)	0 (0.0)		
Hospital length of stay, mean (SD), d	2.4 (4.8)	1.3 (0.9)	1.0 (0.0)	ANOVA	0.54
Follow-up, mean (SD), y	3.4 (2.3)	3.3 (2.2)	3.4 (2.3)	ANOVA	0.89
Reoperation, ***N*** (%)	11 (13.8)	1 (3.8)	0 (0.0)	*X*^2^	0.16

**Table 3:** Epilepsy cohort clinical outcomes

Characteristic	Lesional	Non-lesional	Test used	*P* value
Total procedures, ***N*** (%)	55 (68.8)	25 (31.3)		
Male, ***N*** (%)	30 (54.5)	14 (56.0)	*X*^2^	0.87
Race, ***N*** (%)			Fisher exact^*^	0.790
White	44 (80.0)	21 (84.0)		
Black	3 (5.5)	2 (8.0)		
Asian	2 (3.6)	0 (0.0)		
AI/AN	1 (1.8)	1 (4.0)		
MENA	1 (1.8)	0 (0.0)		
Other	4 (7.3)	1 (4.0)		
Ethnicity			*X*^2^	0.78
Hispanic	23 (41.8)	12 (48.0)		
Non-Hispanic	32 (58.2)	13 (52.0)		
BMI 95–98th pct, ***N*** (%)	12 (21.8)	10 (40.0)	*X*^2^	0.16
SVI, mean (SD)	0.62 (0.25)	0.57 (0.27)	Ind *t*-test	0.38
Age at surgery, mean (SD), y	13.3 (7.6)	17.1 (2.9)	Ind *t*-test	0.018
sEEG	17 (30.9)	23 (92.0)	*X*^2^	< 0.001
Trajectories			*X*^2**^	0.007
1	19 (34.5)	0 (0.0)		
2	26 (47.3)	12 (48.0)		
3	5 (9.1)	10 (40.0)		
4	3 (5.5)	2 (8.0)		
5	2 (3.6)	1 (4.0)		
Lesion type, ***N*** (%)				
MTS	16 (29.1)	-		
PVNH	5 (9.1)	-		
TS	10 (18.2)	-		
FCD	6 (10.9)	-		
Hamartoma	3 (5.5)	-		
Cavernous malformation	4 (7.3)	-		
Pseudoaneurysm	1 (1.8)	-		
Other tumor	10 (18.2)	-		
Region^+^, ***N*** (%)			Fisher exact^***^	< 0.001
Hippocampal	17 (30.9)	21 (77.8)		
Temporal	8 (14.5)	0 (0.0)		
Frontal	16 (29.1)	1 (3.7)		
Insular	1 (1.8)	6 (22.2)		
Parietal	9 (16.4)	0 (0.0)		
Hypothalamus	3 (5.5)	0 (0.0)		
Thalamus	2 (3.6)	0 (0.0)		
Occipital	5 (9.1)	0 (0.0)		
Preoperative seizures/month, mean (SD)	21.9 (33.6)	18.0 (33.6)	Ind *t*-test	0.64
Seizure frequency, mean % change (SD)	−86.6 (29.5)	−78.3 (36.4)	Ind *t*-test	0.30
Total patients, ***N*** (%)	49 (72.1)	19 (27.9)		
Outcome, ***N*** (%)^++^			*X*^2****^	0.66
ILAE 1	28 (57.1)	7 (36.8)		
ILAE 2	1 (2.0)	2 (10.5)		
ILAE 3	4 (8.2)	4 (21.1)		
ILAE 4	8 (16.3)	2 (10.5)		
ILAE 5	5 (10.2)	2 (10.5)		
Unknown	3 (6.1)	2 (8.0)		
Complication, ***N*** (%)	9 (18.4)	1 (4.0)	Fisher exact	0.24
Wound dehiscence	2 (22.2)	0 (0.0)		
Transient weakness	7 (77.8)	0 (0.0)		
Bleed	0 (0.0)	1 (100.0)		
Repeat ablation, ***N*** (%)	5 (10.2)	6 (31.6)	*X*^2^	0.18

*Patient race grouped into white vs. non-white patients

**Trajectories grouped into one and two trajectories vs. more than two trajectories

***Region grouped into hippocampal vs. non hippocampal

****Outcomes grouped into ILAE 1 and ILAE 2 vs. ILAE other outcome

^+^Six lesional patients had lesions in two regions; three non-lesional patients had hippocampal and insular ablation

^++^Outcome is by patient; reoperation patient outcome is only included for the second operation

In the patients with lesional epilepsy, the average age at surgery was 13.3 ± 7.6 years, 12 procedures (21.8%) had a preoperative BMI within the 95th–98th percentile for age, and the average SVI was 0.62 ± 0.25. Most lesional procedures were completed with one (19 (34.5%)) or two (26 (47.3%)) trajectories; lesions were most commonly located in the hippocampus (17 (30.9%)) or frontal lobe (15 (27.3%)). Within the non-lesional group, 23 procedures (92.0%) were performed following pre-ablation sEEG, and 21 procedures (84.0%) underwent hippocampal ablation. In non-lesional patients, most procedures were completed with two (12 (48.0%)) or three (10 (40.0%)) trajectories. The average age at surgery was 17.1 ± 2.9 years; 10 patients (40.0%) had a BMI between the 95th–98th percentile, and the average SVI was 0.57 ± 0.27. Preoperative seizure frequency was 23.7 ± 34.5 in the lesional group, compared to 18.0 ± 33.6 in the non-lesional group (*p* = 0.64).

Significant differences were noted in age at surgery (13.3 ± 7.6 lesional, 17.1 ± 2.9 non-lesional, *p* = 0.02), number of trajectories used (45 lesional procedures completed with one or two trajectories (81.8%), 12 non-lesional (48.0%), *p* = 0.007), ablated region (17 (30.9%) hippocampal lesional procedures, 21 (77.8%) hippocampal non-lesional procedures, *p* < 0.001), and sEEG use, which was present in 17 (30.9%) of lesional procedures and 23 (92.0%) of non-lesional procedures (*p* < 0.001). There were no significant differences between the lesional and non-lesional groups in any baseline demographic characteristics.

**Fig. 1:**
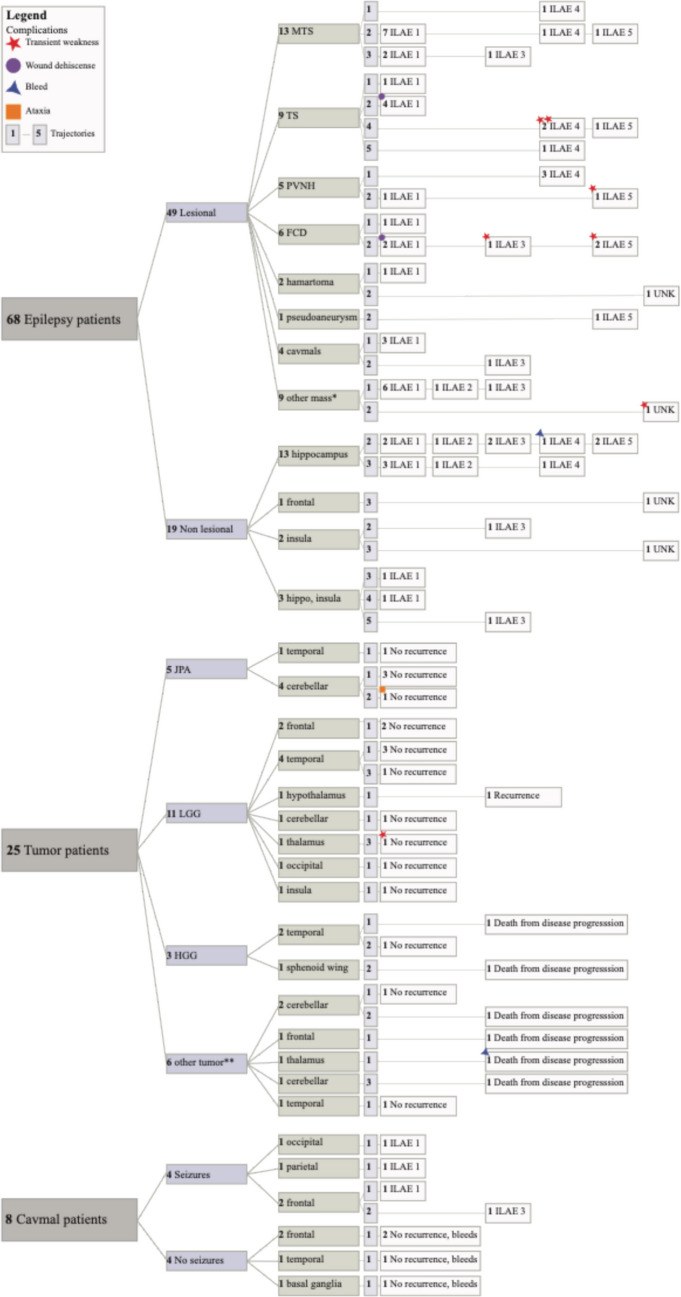
Clinical and outcome characteristics of included participants. Patients are grouped by primary MRgLITT indication (epilepsy, tumor, cavernous malformation). Within each indication, outcomes are displayed by clinically relevant subgroups (e.g., lesion status, pathology, ablation region, and trajectory number). Patients with epilepsy secondary to tumors or cavernous malformations are represented in both epilepsy and tumor/CCM groups to facilitate indication-specific outcome analyses. Outcomes are reported within each indication and are not pooled across disease entities. *Other masses include: 5 LGG, meningioma, embryonal, PXA, and DNET. **Other tumors include: medulloblastoma, hemangioma, ependymoma, PNST, and 2 meningiomas

The tumor group included 25 patients and 26 procedures; patients were sorted into the tumor group if they had a confirmed intracranial neoplasm with or without epilepsy, and thus, patients with epileptogenic tumors were included in both the lesional epilepsy and tumor groups to allow tracking of ILAE outcomes in these patients (Fig. 1, Supplemental Table 1). The mean age of surgery was 11.1 ± 5.4 years and included 14 males (56.0%). Eighteen (72.0%) of patients self-reported white race; 12 patients (48.0%) were Hispanic, and the mean SVI was 0.55 ± 0.26. MRgLITT was performed concurrently with biopsy in 17 of 25 (68%) patients. Tumor histology revealed 5 (19.2%) juvenile pilocytic astrocytomas (JPA), 12 (46.2%) low grade gliomas (LGG), 3 (11.5%) high grade gliomas (HGG), 2 (7.7%) meningiomas, and 1 medulloblastoma, 1 ependymoma, 1 PNST, and 1 hemangioma. Patients in the HGG group were treated with MRgLITT as a salvage therapy in the setting of refractory disease. Most ablated lesions were in the temporal lobe (eight procedures (30.8%)), followed by eight in the cerebellum (30.8%), three frontal (11.5%), one sphenoid wing (3.8%), three thalamus (11.5%), one hypothalamus (3.8%), one occipital (3.8%), and one insula (3.8%). Mean length of follow-up for the tumor group was 3.3 years (range 0.2–6.7). Tumors were measured as the largest cross-sectional length on preoperative MRIs. In this cohort, the mean tumor target size was 16.6 ± 9.4 mm. Most tumors (69.2%) were ablated with one trajectory; however, five tumors (19.2%) required two trajectories, and three (11.5%) required three trajectories. Multiple trajectories were used for large, irregular tumors with satellite lesions or in cases with periventricular, eloquent, or brainstem projections.

The CCM group comprised eight patients (average age at surgery 11.7 ± 6.2 years), including six (75.0%) males, with MRI confirmed CCMs [24] (Fig. 1, Supplemental Table 2). All patients self-reported white race; four (50.0%) identified as Hispanic, and the average SVI was 0.47 ± 0.30. Half of the patients presented with epilepsy, while the remaining patients presented incidentally or with headaches. Patients with incidental presentation were treated following progressive lesion increase on imaging. Half of the ablated lesions were in the frontal lobe. All but one CCM was ablated with one trajectory. One patient required two trajectories due to the presence of distinct epileptogenic regions on preoperative MRI and vEEG, thus necessitating a dual trajectory approach to achieve sufficient ablation. Mean follow-up was 3.4 years (range 0.2–7.0).

### Illustrative case 1: non-lesional epilepsy (Fig. 2)

**Fig. 2:**
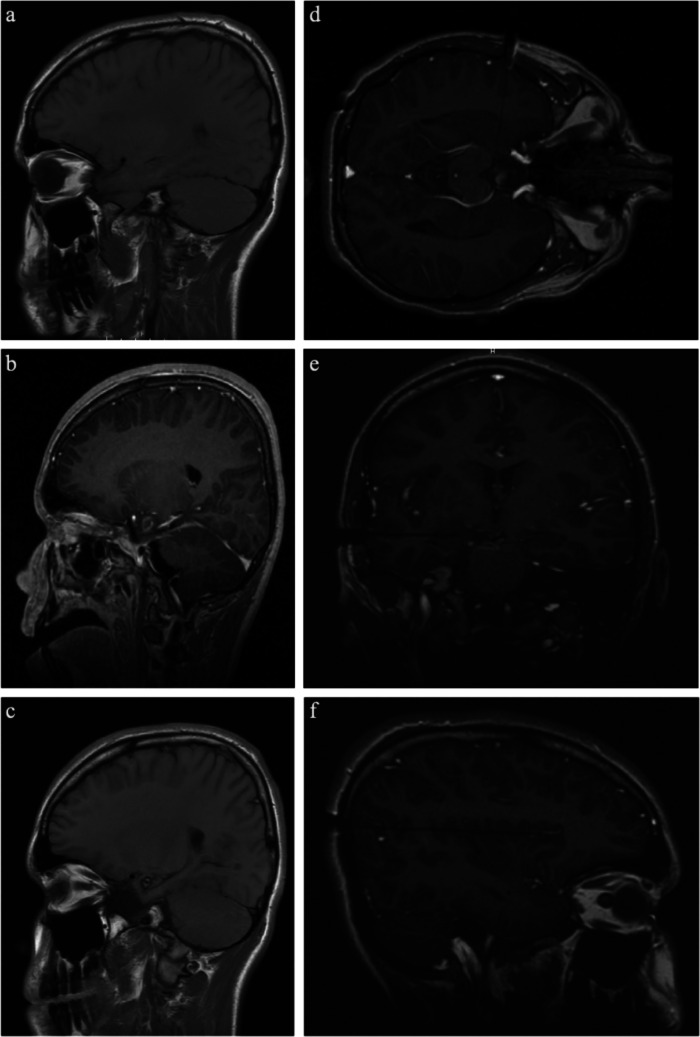
Illustrative case 1: non-lesional epilepsy. **a** Preoperative, **b** immediate postoperative, and **c** 5-year postoperative T2 FLAIR MRI. **d** Amygdala, **e** hippocampal, and **f** superior insular intraoperative trajectories

A 13-year-old boy presented with new onset seizures with quick progression to approximately 14 seizures per month. EEG demonstrated generalized spike and wave discharges; MRI was unremarkable. Following three failed anti-epileptic drugs trials, the patient underwent two admissions for vEEG monitoring, which captured focal right frontotemporal epileptiform discharges. Given the patient’s refractory disruptive epilepsy in the setting of focal and reproducible vEEG findings, he was referred for epilepsy surgery. Surgical planning included placement of eight sEEG leads in the right mesial temporal and insular region, which captured independent onsets from the right insula and hippocampus, for which he was offered MRgLITT treatment. Laser-ablation was planned with a three-trajectory approach, targeting the right superior insula, hippocampus, and amygdala. Follow-up MRI at 1 year post-ablation demonstrated stable ablation changes. He experienced no perioperative complications or deficits and was tapered off all anti-epileptic medications by 2 years and followed for 5 years with no further seizures (Fig. 2).

### Illustrative case 2: thalamic ganglioglioma (Fig. 3)

**Fig. 3:**
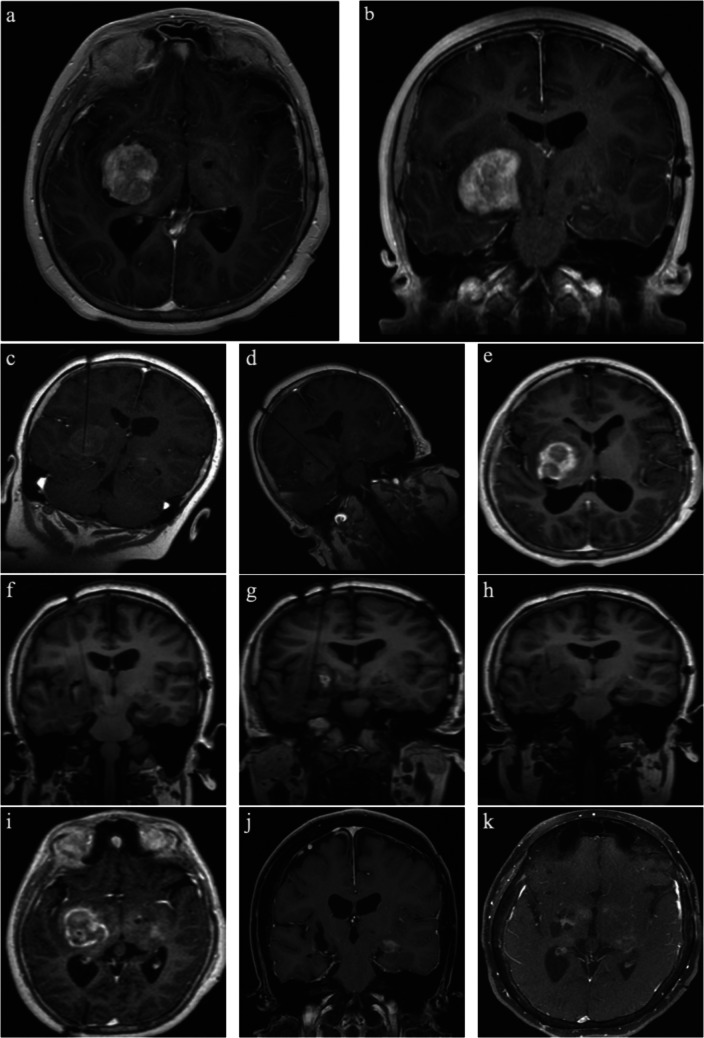
Illustrative case 2: thalamic ganglioglioma. **a**, **b** Preoperative imaging demonstrating right thalamic glioma. **c**, **d** Intraoperative imaging with right anterior and posterior targets. **e** Immediate postoperative MRI. **f**–**h** Intraoperative imaging during reoperation procedure with three trajectories to the mesial, anterior, and posterior thalamic mass. **i** Immediate postoperative imaging following reoperation. **j**, **k** Subsequent follow-up imaging at 7 years following reoperation

This is a 12-year-old female who was diagnosed with bilateral optic pathway gliomas at 5 months of age with multiple stigmata of neurofibromatosis 1 on imaging and negative genetic testing. Despite several courses of cytotoxic chemotherapy, she experienced slow progression leading to loss of vision in her left eye, nearsightedness in her right eye, and left hemiparesis. Due to limited disease control and poor tolerance of medical management, she was offered MRgLITT to reduce tumor burden. Laser ablation was planned with two trajectories for biopsy and ablation of the right anterior and posterior aspects of the thalamic tumor. Postoperatively, she experienced transient worsening of her baseline left sided weakness and improvement in her vision; however, this began to worsen 2 months postoperatively. She was scheduled for reoperation to reduce tumor mass effect on the internal capsule. The operation was planned with three trajectories aimed at the mesial, anterior, and posterior aspects of the thalamic tumor, with reduced ablation power to the mesial target due to the proximity of the internal capsule. She experienced a greater increase in her baseline left sided weakness following her second ablation; however, this improved with rehabilitation. Imaging up to 7 years following her second laser ablation demonstrated no evidence of local recurrence or other disease progression (Fig. 3).

### Illustrative case 3: mesial temporal meningioma (Fig. 4)

**Fig. 4:**
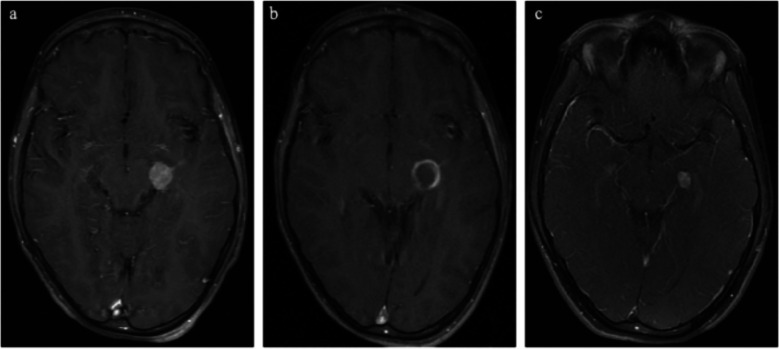
Illustrative case 3: mesial temporal meningioma. **a** Preoperative imaging demonstrating mesial temporal meningioma. **b** Immediate postoperative MRI demonstrating ring-enhancing lesion. **c** Imaging at 5 years post-operation which demonstrates stable, enhancing nodule with no progression

A 6-year-old male who was assessed following an unwitnessed fall down the stairs was found to have a mesial temporal extra-axial mass with surrounding vasogenic edema. He then had a robotic biopsy, and pathology was consistent with meningioma. The patient’s family chose to undergo MRgLITT, and the procedure was planned with one trajectory due to the small size and uniform shape of the lesion. There were no complications, and the patient experienced no neurological deficits. The patient has experienced no seizures and has now tapered off all anti-epileptic medications. Imaging up to 5 years demonstrates a stable, enhancing nodule (Fig. 4).

### Outcome summary

Surgical and outcome details are included in Fig. 1 and Table 3. In the epilepsy cohort, 35 of 68 (51.5%) patients achieved an ILAE 1 outcome. Among tumor patients, the majority (17 of 25 (68.0%)) had no evidence of recurrence at their most recent follow-up. All CCM patients demonstrated symptomatic resolution, and among those with follow-up MRI, six of seven demonstrated radiographic resolution. A total of 13 complications occurred in 12 patients: 10 within the epilepsy group and 3 in the tumor group. Transient weakness was the most common complication, occurring in six patients (one patient had transient weakness following their initial and reoperation) in the epilepsy cohort in expected regions based on ablation location. Other complications included wound dehiscence (two epilepsy procedures), transient ataxia (one tumor patient), and bleeds (one uncomplicated IVH in an epilepsy patient and one bleed from a remote lesion in a tumor patient leading to postoperative death). Of the 13 complications, 9 were minor, requiring short-term physical therapy or simple wound care. Three major complications occurred, including one transient ataxia which required prolonged physical therapy or inpatient rehabilitation and two bleeds which required ICU care, and one of which led to death. Hospital length of stay was short across groups, with no significant differences between indications: epilepsy 2.3 ± 4.7 days, tumor 1.2 ± 0.9 days, and CCM 1.0 ± 0.0 days. Postoperative follow-up length was also similar: epilepsy 3.4 ± 2.3 years, tumor 3.3 ± 2.2 years, and CCM 3.4 ± 2.3 years.

There was no significant difference in ILAE outcome or percent reduction in seizures per month between the lesional and non-lesional cohorts. At most recent follow-up, groups had a similar proportion of patients with an excellent (ILAE 1) outcome: 28 of 49 (57.1%) in the lesional group and 7 of 19 (36.8%) in the non-lesional group. Within the lesional group, surgical outcome differed by type of lesion. Patients with CCMs (3 of 4 (75%)) and MTS (9 of 13 (69.2%)) had the highest rates of ILAE 1 outcome. In comparison, only five of ten TS patients (50%), three out of six FCD patients (50%), and one of five PVNH patients (20%) resulted in an ILAE 1 outcome.

**Fig. 5:**
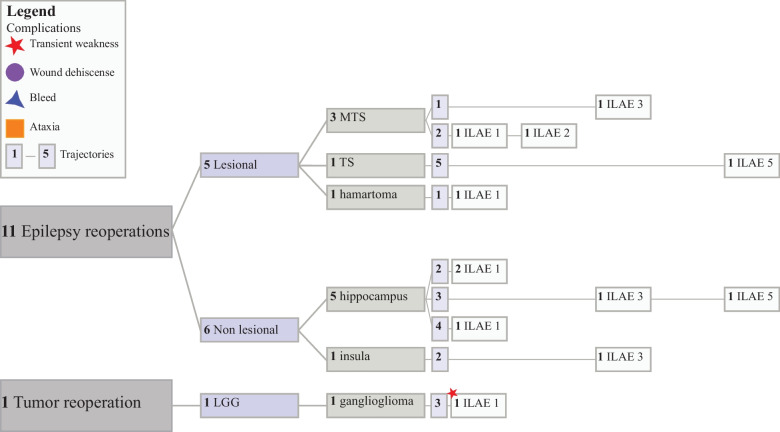
Summary of reoperation cases. Eleven epilepsy patients and one tumor patient required reoperation. Among epilepsy patients, five lesional and six non-lesional patients required reoperation. One LGG patient required reoperation and achieved ILAE 1 outcome

Repeat ablation details are included in Fig. 5. Reoperation was required in 11 patients in the epilepsy group and 1 in the tumor group. Although a greater proportion of non-lesional patients (6 of 19 (31.6%)) underwent reoperation compared to lesional patients (5 of 49 (10.2%)), there was no significant difference between the groups in eventual outcome (*p* = 0.17). Three non-lesional reoperation procedures (50.0%) achieved an ILAE 1 outcome, two had substantial seizure reduction (ILAE 3), and one had modest seizure control (ILAE 5). In the lesional cohort, three of six patients (MTS, HH, LGG) were completely seizure free following reoperation (ILAE 1).

To identify predictors associated with postoperative seizure outcome and reoperation, we performed multivariable logistic regression using clinically prespecified models. ILAE outcome was dichotomized as favorable seizure control (ILAE 1–2) versus unfavorable outcome (ILAE 3–5). Covariates included lesion status on MRI, number of trajectories (1–2 vs. ≥ 3), follow-up duration, and SVI, which was standardized (z-score) prior to analysis. In the adjusted model, higher SVI was independently associated with lower odds of achieving an ILAE 1–2 outcome (OR per 1-SD increase = 0.49, 95% CI 0.27–0.89, *p* = 0.020). No other covariates were significantly associated with seizure outcome. In the reoperation model, lesional epilepsy was associated with lower odds of reoperation, although this did not reach statistical significance (OR 0.25, *p* = 0.06).

We assessed tumor outcomes overall and stratified by tumor type and location **(**Fig. 1, Supplemental Table 1). At a mean follow-up of 3.3 ± 2.2 years, 18 of 25 patients (72.0%) demonstrated no evidence of local recurrence. One patient with LGG (4.0%) experienced local recurrence requiring craniotomy, and one additional LGG patient (4.0%) required repeat ablation. Five patients (20.0%) achieved initial radiographic control with symptomatic improvement but subsequently died due to distant disease progression. One patient (4%) experienced post-ablation hemorrhage from a distant lesion and died on post-ablation day 1 (total deaths *n* = 6).

Postoperative complications occurred in 3 of 25 patients (12.0%). One patient experienced transient postoperative weakness that improved with outpatient physical therapy, and one experienced post-ablation ataxia requiring inpatient rehabilitation with subsequent improvement. One patient with a PNST underwent MRgLITT as salvage therapy in the setting of progressive disease and experienced hemorrhage from a distant lesion, resulting in death on the first postoperative day.

Tumor outcomes varied by diagnosis. All patients with JPA, 8 of 12 patients (66.7%) with LGG, and 1 of 3 patients with HGG demonstrated no local recurrence. One patient with a cerebellar hemangioma had no recurrence, while the remaining four patients demonstrated no local recurrence following ablation but ultimately died from disease progression. Overall, disease progression leading to death occurred in six patients, including one medulloblastoma, one ependymoma, one PNST, two HGG, and one meningioma.

Eight patients underwent MRgLITT for CCMs (Fig. 1, Supplemental Table 2). Patients experienced no perioperative complications. Among the patients with seizures, three of four achieved an ILAE 1 outcome. The remaining patient (ILAE 3) experienced postoperative seizures only in the setting of missed AEDs. Seven patients had follow-up MRIs, of which six demonstrated no evidence of residual lesion, growth, or interim bleeds.

## Discussion

MRgLITT is an established option for adult intracranial pathologies, but evidence in pediatric populations remains limited. This study presents one of the largest pediatric MRgLITT series to date, demonstrating high rates of seizure control, tumor stability, and radiographic resolution across diverse indications with a low complication rate. Our findings support MRgLITT as a safe, effective, and repeatable treatment for children with epilepsy, tumors, and cavernous malformations.

### Epilepsy

In children with lesional DRE, laser ablation has been increasingly adopted as a minimally invasive alternative to surgical resection. While resection is associated with favorable outcomes (53–84% seizure freedom) [25] multiple studies report that only a fraction of eligible patients are referred for surgical evaluation due to system barriers including limited access, low referral rates, and lack of patient education [26, 27]. Even among referred patients, significant delays between DRE diagnosis and surgical resection are common [28], and patients with lower income are less likely to access epilepsy surgery [29]. Compared to medical treatment, surgical treatment of pediatric DRE is associated with improved survival, reduced healthcare utilization, and fewer AEDs, thus indicating the need for wide adoption of minimally invasive techniques as an initial surgical option [30].

Laser ablation for the treatment of pediatric lesional DRE has been described [8, 10]. However, limited data [17] exist detailing the safety and efficacy of MRgLITT for non-lesional pediatric DRE. Non-lesional epilepsy is more challenging to treat surgically; reports in adults show two to three times higher seizure freedom rates in patients with a lesion on MRI [31]. In our pediatric DRE cohort, we observed no significant difference in postoperative ILAE outcomes between groups. However, non-lesional patients were more likely to require both preoperative sEEG and repeat ablation, undergoing two additional procedures compared to lesional epilepsy patients. Despite additional required operations, reoperation did result in seizure freedom (ILAE 1) for half of reoperation procedures. Within the entire epilepsy cohort, reoperation was not associated with worse ILAE outcome, suggesting MRgLITT is successful in procedures which require reoperation and is also effective for patients with non-lesional epilepsy.

There was notable heterogeneity in lesion type treated within the lesional group. The most common lesion identified was MTS. These patients did well after ablation; over half of the patients in the MTS group achieved an ILAE 1 outcome. In contrast, patients with PVNH experienced worse outcomes, with only one of five reaching ILAE 1. Lower rates of seizure freedom among PVNH patients are likely associated with the diffuse, multifocal nature of PVNH, leading to challenges in effective treatment with focal ablation [32]. Although conclusions are limited by the sample size of five procedures, the patient who did achieve seizure freedom was treated with two trajectories, suggesting that multiple trajectories may be beneficial for effective treatment of this lesion.

Previous literature described many predictors of seizure freedom following epilepsy surgery, including the presence of a lesion on preoperative MRI, identified MTS, EEG/MRI concordance [33] and socioeconomic status [34]. In our study, higher SVI was associated with worse clinical outcome; in the reoperation model, non-lesional epilepsy trended toward higher reoperation rates; however, this was not statistically significant (*p* = 0.06). Social vulnerability reflects the resource availability in the patient’s geographical environment and is an imperfect measure of socioeconomic status. Nonetheless, it has been shown to predict poorer recovery in other surgical studies [35] and is associated with worse outcomes in our cohort. This could be due to several important factors. Epilepsy surgery outcome is not only due to surgical intervention but also related to postoperative care, recovery support systems, and reliable access to AEDs. These factors are particularly crucial in pediatric patients who rely on caregiver support, and therefore, these factors may have a larger impact on ultimate recovery and seizure freedom rates following MRgLITT.

As described above, surgical treatment of non-lesional epilepsy is associated with lower rates of seizure freedom. In our analysis, we found no significant differences in ILAE outcome or reoperation rate between lesional and non-lesional epilepsy groups, although we did observe an increased rate of reoperation in the non-lesional group. This finding suggests that while reoperation may be required at a higher rate in non-lesional patients, reoperation can lead to eventual seizure freedom and MRgLITT is a therapy amenable to reoperation. While important, these findings are in small patient cohorts with imperfect measures and are therefore challenging to generalize.

### Tumors

While first introduced in pediatric neurosurgery for the treatment of brain tumors, and widely used in adult primary and metastatic brain tumors [36, 37], pediatric outcome data remain scarce [9, 38]. In pediatric intracranial neoplasms, MRgLITT is particularly suitable due to its versatility: ablation can be done in patients of any age and preoperative functional score, can be repeated in cases of tumor recurrence, and can easily be done in conjunction with other surgical treatments and biopsy. Prior reports note that MRgLITT is often selected for tumors which are challenging to identify intraoperatively, are deep seated, or for recurrent disease [9]. Other notable reasons to choose MRgLITT include the treatment of relatively small tumors, where focal ablation is feasible and may lead to fewer complications, as seen in our cohort.

In our series, 72.0% of tumor patients had no recurrence following laser ablation. An additional 24.0% underwent laser ablation for palliative symptom control in the setting of advanced disease, and while only one patient experienced recurrence in the local ablation cavity, these patients ultimately died due to progressive disease. Although complication rates were lower than those reported in prior series (3 of 25 patients), two of these events were major, including one requiring prolonged inpatient rehabilitation and one resulting in postoperative death due to distant hemorrhage, underscoring that serious complications, while uncommon, may occur following MRgLITT. Hospitalization length was also shorter compared to prior studies; most were discharged 1 day postoperatively; two patients required hospitalization for 2 days, one for 3 days, and one for 5 days.

### Cavernous malformations

MRgLITT is an alternative surgical management for cavernous malformations, enabling precise ablation to both provide a sole treatment option for patients with challenging to reach lesions. Despite its potential, there are only a total of 11 patient reports of MRgLITT in pediatric CCMs [6, 39, 40]. Small cohort sizes substantially limit generalizability; however, existing data demonstrates high seizure freedom rates, with between 80 and 90% of patients reaching Engel I and no instances of re-hemorrhage. Our findings, while among a small number of patients, are consistent with these favorable outcomes: high rates of seizure freedom with no complications and short hospital stays over an average 3.4-year follow-up. Three of four patients achieved an ILAE 1 outcome. The remaining patient had seizures only in the setting of missed AEDs. One patient underwent ablation using two trajectories without recurrence or complications, demonstrating the utility of MRgLITT in ablating large and irregular CCMs. Our results add to the growing body of literature supporting MRgLITT as a safe and efficacious treatment for pediatric CCMs.

### Limitations

This study has limitations inherent to retrospective analyses, including errors in electronic charting, selection biases, and its single-center, single-surgeon design, with no control group. Prospective randomized control trials comparing MRgLITT to resection would be ideal to demonstrate clinical superiority and identify which patients and indications may benefit most. However, as a noted advantage of MRgLITT is the ability to treat patients who are unable to undergo resection, a prospective study design is unlikely to be clinically relevant. In the epilepsy group, this study does not control for varied AED regimens and duration of epilepsy prior to treatment. Other limitations include small sample sizes within indication subgroups, limiting our ability to derive statistically significant conclusions and a variable follow-up period. While the overall average follow-up time was substantial, some patients had limited follow-up due to travel constraints. Future directions will require multicenter, prospective trials comparing MRgLITT to standard of care in pediatric patients with standardized follow-up periods.

## Conclusions

MRgLITT is an effective treatment modality with demonstrated effectiveness in the treatment of intracranial pathology in adults [36, 37, 41]. However, studies evaluating its use in pediatric populations remain sparse. The development of minimally invasive neurosurgical treatment options is of particular interest in pediatric populations, where offering timely intervention and reducing postoperative morbidity is especially important. In this cohort, we demonstrate effective treatment across a variety of pediatric indications with low complication rates. Half of all epilepsy patients achieved complete seizure freedom, and seizure outcomes were similar for lesional and non-lesional cohorts, with repeat ablation restoring control in several refractory patients. Seventeen of 25 tumor patients experienced no local recurrence after a mean 3-year follow-up, while all cavernous-malformation patients achieved symptomatic resolution. Major complication rates were low (3%), and median hospital stay was limited to 1 day. Multivariable analysis identified higher SVI as a predictor of poorer seizure outcome, underscoring the importance of perioperative support systems. These data support the adoption of MRgLITT as a first-line option in the treatment of various pediatric intracranial pathologies.

## Supplementary Information

Below is the link to the electronic supplementary material.

ESM 1 Supplementary Material 1 (DOCX 18.9 KB)

## Data Availability

No datasets were generated or analysed during the current study.
