# Supplementary material for: MRI-guided laser ablation for pediatric intracranial pathology: single center experience
Source: Childs Nerv Syst. 2026 Jan 24;42(1):44. doi: 10.1007/s00381-026-07133-y (PMC12830497; doi:10.1007/s00381-026-07133-y)
Supplement: Supplementary file 1 — Supplementary Material 1 (DOCX 18.9 KB) [file 381_2026_7133_MOESM1_ESM.docx]

**Supplemental Material**

Supplemental Table 1: Tumor Cohort Clinical Outcomes

| Characteristic |  |
| --- | --- |
| Total cases, N | 26 |
| Concurrent biopsy | 17 (68.0) |
| Tumor type, N (%) |  |
| JPA | 5 (19.2) |
| LGG | 12 (46.2) |
| HGG | 3 (11.5) |
| Meningioma | 2 (7.7) |
| Medulloblastoma | 1 (3.8) |
| Ependymoma | 1 (3.8) |
| PNST | 1 (3.8) |
| Hemangioma | 1 (3.8) |
| Location, N (%) |  |
| Temporal | 8 (30.8) |
| Cerebellum | 7 (26.9) |
| Frontal | 3 (11.5) |
| Thalamus | 3 (11.5) |
| Posterior Fossa | 2 (7.7) |
| Occipital | 1 (3.8) |
| Insular | 1 (3.8) |
| Hypothalamus | 1 (3.8) |
| Target size*, mean (SD), mm | 16.6 (9.4) |
| Total patients, N (%) | 25 |
| Outcome |  |
| No recurrence | 17 (68.0) |
| Local recurrence | 2 (8.0) |
| Death from disease progression | 6 (24.0) |
| Reoperation, N (%) | 1 (3.8) |

*Target size measured as largest cross section

Supplemental Table 2: Cavernous Malformation Cohort Clinical Outcomes

| Characteristic |  |
| --- | --- |
| Total, N | 8 |
| Location, N (%) |  |
| Frontal | 4 (50.0) |
| Insula | 1 (12.5) |
| Lentiform nucleus | 1 (12.5) |
| Occipital | 1 (12.5) |
| Parietal | 1 (12.5) |
| Outcome |  |
| Complication | 0 (0.0) |
| Resolved | 8 (100.0) |
| Recurrence | 0 (0.0) |
| Epilepsy, N (%) | 4 (50.0) |
| Epilepsy Outcome |  |
| ILAE 1 | 3 (75.0) |
| ILAE 2 | 0 (0.0) |
| ILAE 3 | 1 (25.0) |
| ILAE 4 | 0 (0.0) |
| ILAE 5 | 0 (0.0) |
